# Balloon sinuplasty: does a model simulator improve ENT trainee competence?

**DOI:** 10.1308/rcsann.2024.0075

**Published:** 2025-10-21

**Authors:** R Pinto, R Sharma, G Khong

**Affiliations:** Alder Hey Children’s NHS Foundation Trust, UK

**Keywords:** Sinusitis, Frontal sinus, Simulation training

## Abstract

**Introduction:**

Balloon sinuplasty is an established technique for treating chronic rhinosinusitis and its role in managing complicated acute rhinosinusitis (ARS) is emerging. Complicated ARS is a potentially life-threatening condition and balloon sinuplasty is not currently a competency required by UK otolaryngology trainees. To our knowledge, we are the first to investigate whether a balloon sinuplasty model simulator was able to improve trainee self-perceived competence.

**Methods:**

A prospective, qualitative-based study was carried out including all candidates attending a national paediatric course rotating through our balloon sinuplasty model simulator station. Candidates returned a questionnaire to evaluate their experience.

**Results:**

Twenty feedback forms were returned. Candidates ranged from core trainees to consultants, with the majority being registrar or senior clinical fellow level (89.4%). Three candidates had previously performed balloon sinuplasty in adults and none in children. The number of candidates rating their understanding of how to perform balloon sinuplasty as ≥7 increased after the simulator experience from 9 of 20 (45%) to 20 of 20 (100%). Following the model simulator experience, 45% felt confident in performing balloon sinuplasty in adult patients and 25% in children. In all, 40% of candidates were confident in using balloon sinuplasty electively and 35% felt they would be able to use it in an emergency procedure.

**Conclusions:**

Surgical confidence in performing balloon sinuplasty is generally low, especially among trainees. A balloon sinuplasty model simulator is able to improve this but further training and hands-on experience is required to facilitate translation of simulation acquired surgical skills to the operating theatre.

## Introduction

Chronic rhinosinusitis (CRS) is a common condition affecting 11% of the population and significantly affects patient quality of life.^1,^^2^ The frontal sinus has been shown to be involved in CRS in 90.2% of cases with nasal polyposis and 82.6% in those without.^3^ Outcomes following functional endoscopic surgery (FESS) for chronic frontal sinusitis are favourable.^4^ However, the frontal sinus is a notoriously challenging area in which to surgically intervene owing to the complex anatomy of the frontal recess. For this reason the frontal sinus is usually tackled by expert rhinologists and anterior skull base surgeons.^5^ Potentially as an indirect result, frontal sinus surgery in England accounts for only 12.5% of all endoscopic sinus procedures.^6^

Balloon sinuplasty has been shown to be a safe and effective surgical procedure for draining the paranasal sinuses in both adults and children.^7,^^8^ In particular, success rates and patient satisfaction following frontal sinus balloon sinuplasty are high.^9^ Compared with FESS, balloon sinuplasty is technically less challenging, more cost-effective and carries a lower complication rate.^10^

In the acute setting, sinusitis may be complicated by orbitocranial extension of infection resulting in suppuration and abscess formation, which may threaten vision and even life.^11^ These complications are relatively uncommon but have been shown to occur in 3.7%–20% of patients with sinusitis.^12^ The frontal sinus is the most frequently involved sinus in acute sinugenic intracranial infection and can also result in orbital abscess or Potts puffy tumour.^13^ Traditionally, management of complicated acute frontal sinusitis involves intravenous antimicrobials and surgical drainage, which may be carried out by either frontal sinus trephination or FESS.^14^ Drawbacks of the current surgical approaches mainly include facial skin incision for frontal sinus trephination and technical difficulty in performing FESS in an acutely hyperaemic surgical field. Furthermore, evidence is emerging in the literature that balloon sinuplasty may be a safe and effective alternative surgical strategy to current approaches.^15,16^

The data currently suggest that balloon sinuplasty may have a significant role to play in the acute and elective management of sinusitis. We believe otolaryngology trainees would benefit from training for this procedure as part of their surgical repertoire. We investigated whether a balloon sinuplasty model simulator improved trainee knowledge and confidence in performing this procedure in the operating room.

## Methods

### Ethical considerations

This study was undertaken according to our trust’s audit department protocol. The manufacturer Stryker did not have any role in the design, analysis or write up of this article.

### Model simulator design

**Figure 1:**
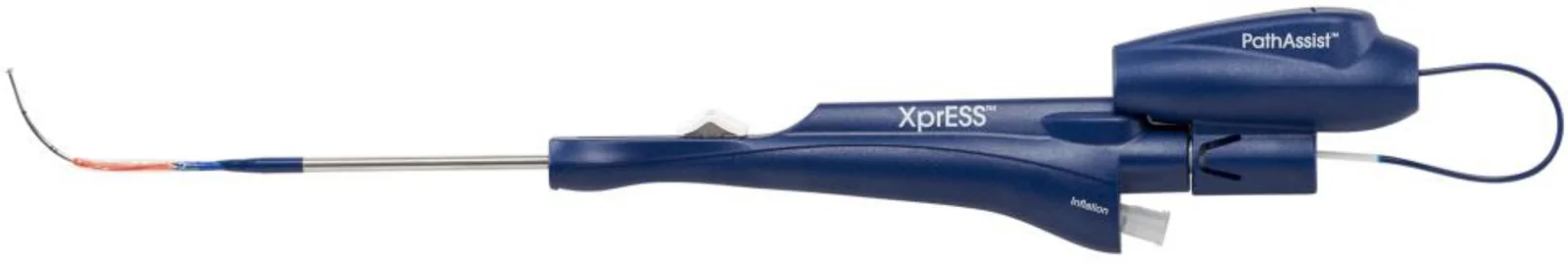
The XprESS Balloon (6mm × 20mm LPLF-106-I) by Stryker^©^

A model created by Stryker^©^ using the XprESS Balloon (6mm × 20mm LPLF-106-I) (Figure 1) designed specifically for balloon sinuplasty training was implemented in this study. The model represented an adult-size head and sinuses. The nasal cavities, paranasal sinuses, eustachian tubes and nasopharynx were accessible through the anterior nares with a rigid nasal endoscope. The simulator was designed to pass the balloon into the frontal recess to recreate a surgical frontal sinusotomy. Two operators were required for the simulation. The primary operator handled the rigid endoscope and balloon. The secondary operator handled the syringe used for inflating the balloon. Balloon placement confirmation was achieved using the balloon tip fibreoptic light with subsequent sinus cavity illumination. The plunger on the syringe was depressed and the balloon inflated after confirming adequate placement with a fibreoptic light at the tip.

### Population and setting

A national practical paediatric course was hosted by Alder Hey Children’s Hospital in July 2022. The purpose of this course was to provide candidates with hands-on practical experience across a wide range of procedures carried out within the scope of paediatric otolaryngology. Candidates included doctors with a varied level of experience including all grades between core surgical training and consultancy. One of the stations at this course offered training in balloon sinuplasty using the Stryker^©^ model and balloon described above.

### Study design and data analysis

Course candidates who rotated through our balloon sinuplasty station were first provided with a detailed combined verbal and practical demonstration provided by consultant otolaryngologists and Stryker^©^ representatives, followed by supervised training on the models. Candidates were specifically taught the component parts of the balloon equipment, preparation of equipment including priming the syringe and technique as described above.

Candidates were then asked to reflect on their experience and answer a six-point questionnaire (Appendix 1 — available online) to evaluate their perceived competence in performing balloon sinuplasty. The questionnaire was delivered via Google Forms accessed using a QR code provided during the station. The data were interpreted for analysis.

### Outcome measures

The outcome measures of this study assessed change in knowledge and confidence related to performing balloon sinuplasty following training on the model simulator. Questions designed to obtain these data did so using a 10-point Likert scale. A score of 7 out of 10 or greater to indicate a successful outcome was deemed to be reasonable by the authors.

## Results

Twenty feedback forms for the model simulator were returned. Candidates participating ranged from core trainees to consultants with 16 of 19 (89.4%) designated registrar or senior clinical fellow level. One candidate did not state their training grade.

Only 3 of 20 (15%) candidates had previously performed balloon sinuplasty in adults, whereas none had done so in children. The number of candidates rating their understanding of how to perform balloon sinuplasty as ≥7 increased after the simulator experience from 9 of 20 (45%) to 20 of 20 (100%). Before the training exercise the lowest observed response was 2 out of 10 compared with 7 out of 10 afterwards.

**Figure 2:**
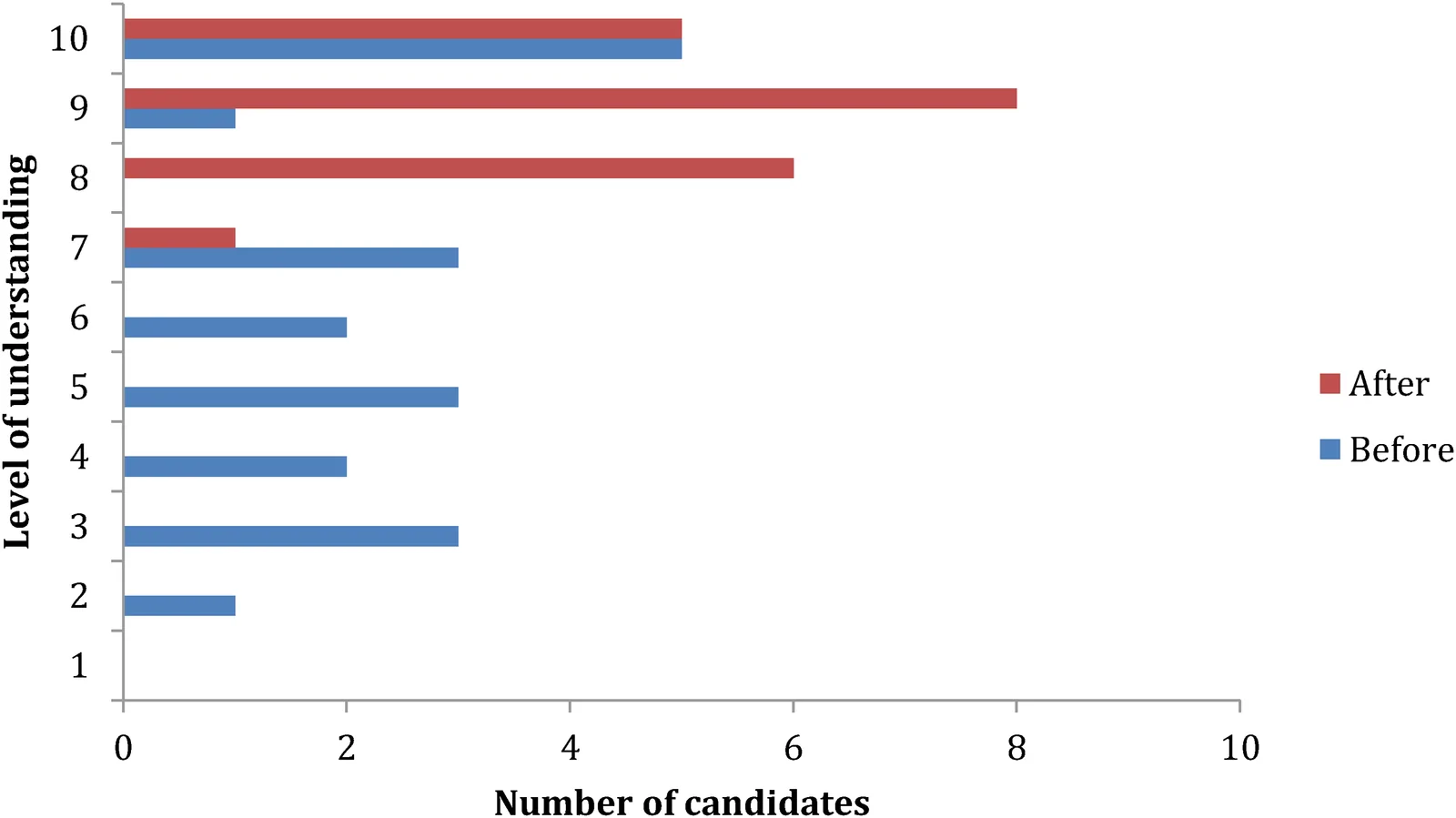
Candidates’ understanding of how to perform balloon sinuplasty before and after the training station

**Figure 3:**
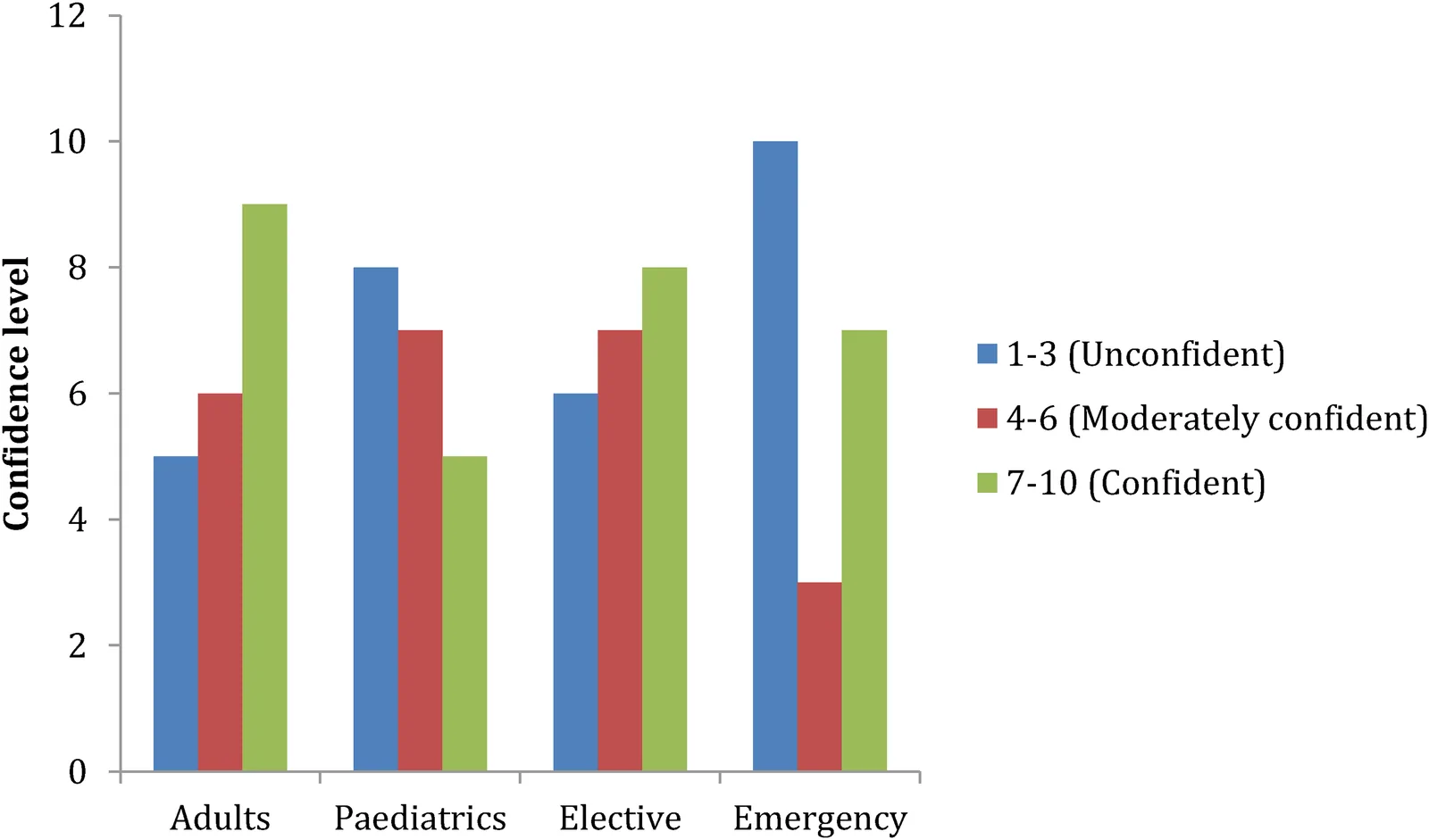
Candidate confidence in performing balloon sinuplasty in adults (a), children (b), an elective case (c) and an emergency case (d)

Following the model simulator experience, 9 of 20 candidates (45%) felt confident in performing balloon sinuplasty in adult patients and 5 of 20 (25%) in children. Similarly, 8 of 20 (40%) candidates felt confident to use balloon sinuplasty electively and 7 of 20 (35%) felt they would be able to use it in an emergency procedure (Figures 2 and 3).

## Discussion

### Synopsis of key findings

Candidates on our course were reasonably inexperienced with regards to performing balloon sinuplasty, especially in children. Cerebral understanding of the procedure increased following model simulation training. Confidence in applying the newly acquired skills in the operating theatre is still lacking.

### Study strengths

We are the first to specifically investigate the use of a model simulator to improve trainee self-perceived competence in balloon sinuplasty. This is an important study because balloon sinuplasty can be used in potentially life-threatening scenarios involving frontal sinusitis, and our data suggest that trainees have relatively little exposure to balloon sinuplasty during their training.

Model simulator training took place as part of a national course attracting otolaryngologists from across the country and some from abroad, and we assume this will provide a reasonable representation of trainee experience from a variety of training backgrounds.

The majority of course candidates included in the study were senior otolaryngology doctors in training. This is arguably the most appropriate population to capture because they are the trainees most likely to benefit from balloon sinuplasty training and application of it to the clinical setting.

A detailed verbal and practical demonstration was provided to the candidates before the simulation as well as consultant-led supervision during the training exercise to optimise candidates’ productivity on the model simulator. This potentially avoids the passive and superficial process of instrumenting the balloon within the model, and hopefully encourages better understanding of the sinus anatomy and appreciation of the technique to employ.

The outcome measures were designed to assess change in knowledge of and confidence in performing balloon sinuplasty, which we felt were clinically applicable.

### Study limitations

Our course was paediatric in nature potentially attracting only those interested in paediatric practice. We had small study numbers, potentially preventing data extrapolation. We had a diverse group of candidates with varying amounts of experience, introducing confounding factors into the data.

Performing sinus surgery in children differs from that performed in adults because of the reduced size of the nasal ethmoid cavities, concave middle meatus and degree of pneumatisation of the cells. This was not reflected in our model simulator, which probably more closely resembles the anatomy of an adult. Therefore, direct correlation of results to the paediatric population may not be possible. A further model with specific paediatric anatomical considerations may improve application of surgical skills to paediatric practice.

We used a non-validated questionnaire for this study. It was intentionally non-exhaustive to prevent trainee burnout during the full-day course, limiting the amount of data we could capture. The questions were designed to be simple while acquiring the relevant data related to the primary outcome measures. Ten-point Likert scales were used to assess our candidates to enable quantifiable data analysis. However, we omitted the use of free-text boxes, which may have provided further insight into candidates’ experience and limitations with balloon sinuplasty. During analysis, we have assumed that a score of 7 out of 10 or greater on the Likert scale is representative of a positive outcome.

### Comparison with other studies

Only one other study has investigated a model simulator for the use of balloon sinuplasty training.^17^ However, the study was different from ours in that it utilised a single expert FESS surgeon operating with the Reliva Sinus Balloon Catheter System to assess whether four different previously validated models could be used for surgical training in balloon sinuplasty. In comparison, our study evaluated how effective our model was in preparing otolaryngology trainees for performing balloon sinuplasty in a non-simulated environment in a variety of acute and elective scenarios.

Model simulation for rhinologic surgical training has been widely studied in the literature for decades. Models range from synthetic to cadaveric. Fried *et al* investigated their model using a prospective multicentre, blinded, control study comparing model-trained vs non-model-trained residents.^18^ In vivo procedures were recorded and assessed by blinded panel experts. The model-trained residents demonstrated statistically significant improved surgical skill, confidence and time to completion with a reduced number of mistakes. Our study design differed significantly in that it was qualitative in nature to gauge trainee perspectives compared with Fried’s study that used objective evidence to establish trainee improvement with model simulation.

### Clinical applicability of the study

CRS is a common condition and complicated acute rhinosinusitis is potentially fatal. General and specialist otolaryngologists encounter both of these conditions. Compared with endoscopic sinus surgery, balloon sinuplasty has been shown to be a comparatively safer and easier procedure to perform, but still effective in draining the paranasal sinuses. Evidence is emerging in the literature regarding its use in complicated acute rhinosinusitis, which can be life-threatening.

Our study demonstrates that trainee otolaryngologists are not experienced or confident in performing balloon sinuplasty despite the common occurrence of sinus disease and the effectiveness of the procedure. Reasons for this may include lack of exposure, training and consultants performing the procedure. We therefore feel that there is a need for trainees to be competent in performing balloon sinuplasty to be able to utilise this surgical tool in both the elective and emergency setting within a secondary or tertiary centre.

Our study demonstrates that the model simulator was able to effectively improve candidates’ knowledge of the procedure from 45% to 100%. However, surgical confidence was still below 50% after training. We may be able to increase confidence further by allowing more time for candidates to explore the model simulator and incorporating balloon sinuplasty in sinus dissection courses. However, ultimately there may be factors leading to reduced surgical confidence that are out of our control such as general lack of experience, especially due to the COVID-19 pandemic.^19,20^ Furthermore, exposure may be limited if balloon sinuplasty is only being performed by a few surgeons in the region in specific departments. Ideally, trainees should have access to the model simulator while working in a department performing balloon sinuplasty regularly to truly extract the maximum learning potential from the training exercise.

## Conclusions

Balloon sinuplasty is a safe and effective method to drain the paranasal sinuses in both adults and children in the emergency and elective settings. Otolaryngology trainees would therefore find this a useful addition to their surgical repertoire. Our model simulator for training in balloon sinuplasty is effective in improving trainee knowledge of the procedure but surgical confidence is still lacking. Further training and hands-on experience to improve translation of balloon sinuplasty knowledge and skill acquired in the simulated setting to the operating theatre is required.

## References

[1] Hastan D, Fokkens WJ, Bachert C *et al.* Chronic rhinosinusitis in Europe–an underestimated disease. A GA^2^LEN study. *Allergy* 2011; **66**: 1216–1223. 21605125 10.1111/j.1398-9995.2011.02646.x

[2] Soler ZM, Wittenberg E, Schlosser RJ *et al.* Health state utility values in patients undergoing endoscopic sinus surgery. *Laryngoscope* 2011; **121**: 2672–2678. 22034223 10.1002/lary.21847 PMC3232034

[3] Sumaily I, Alarifi I, Alahmari A *et al.* Sphenoid sinus involvement in chronic rhinosinusitis without polyps. *Allergy Rhinol (Providence)* 2020; **11**: 2152656720934472. 32596024 10.1177/2152656720934472 PMC7298207

[4] DeConde AS, Smith TL. Outcomes after frontal sinus surgery: an evidence-based review. *Otolaryngol Clin North Am* 2016; **49**: 1019–1033. 27450618 10.1016/j.otc.2016.03.024 PMC4964596

[5] Chen PG, Wormald P-J, Payne SC *et al.* A golden experience: fifty years of experience managing the frontal sinus. *Laryngoscope* 2016; **126**: 802–807. 26393824 10.1002/lary.25648

[6] Gupta KK, Jolly K, Bhamra N, Osborne MS, Ahmed SK. The evolution of sinus surgery in England in the last decade – an observational study. *World J Otorhinolaryngol - Head Neck Surg* 2021; **7**: 240–246. 34430832 10.1016/j.wjorl.2020.10.002 PMC8356113

[7] Castro A, Furtado M, Rego Â *et al.* Long term outcomes of balloon sinuplasty for the treatment of chronic rhinosinusitis with and without nasal polyps. *Am J Otolaryngol* 2021; **42**: 102825. 33202329 10.1016/j.amjoto.2020.102825

[8] Mirza AA, Shawli HY, Alandejani TA *et al.* Efficacy and safety of paranasal sinus balloon catheter dilation in pediatric chronic rhinosinusitis: a systematic review. *J Otolaryngol - Head Neck Surg = Le J d’oto-rhino-laryngologie Chir cervico-faciale* 2020; **49**: 69. 10.1186/s40463-020-00463-0 PMC7523047 32993786

[9] Sainio S, Blomgren K, Koskinen A, Lundberg M. Frontal sinus balloon sinuplasty-patient satisfaction and factors predicting reoperation. *OTO Open* 2023; **7**: e23. 36998566 10.1002/oto2.23 PMC10046702

[10] Bolger WE, Brown CL, Church CA *et al.* Safety and outcomes of balloon catheter sinusotomy: a multicenter 24-week analysis in 115 patients. *Otolaryngol Neck Surg* 2007; **137**: 10–20. 10.1016/j.otohns.2007.02.006 17599558

[11] Germiller JA, Monin DL, Sparano AM, Tom LWC. Intracranial complications of sinusitis in children and adolescents and their outcomes. *Arch Otolaryngol Neck Surg* 2006; **132**: 969–976. 10.1001/archotol.132.9.969 16982973

[12] Fokkens WJ, Lund VJ, Mullol J *et al.* EPOS 2012: European position paper on rhinosinusitis and nasal polyps 2012. A summary for otorhinolaryngologists. *Rhinology* 2012; **50**: 1–12. 22469599 10.4193/Rhino12.000

[13] Remmler D, Boles R. Intracranial complications of frontal sinusitis. *Laryngoscope* 1980; **90**: 1814–1824. 7432063 10.1288/00005537-198011000-00009

[14] Hallak B, Bouayed S, Ghika JA *et al.* Management strategy of intracranial complications of sinusitis: our experience and review of the literature. *Allergy Rhinol (Providence)* 2022; **13**: 21526575221125030. 10.1177/21526575221125031 PMC9513574 36177149

[15] Chidambaram S, Wahle BM, Leonard DS. Frontal balloon sinuplasty in complicated acute pediatric rhinosinusitis (ARS). *Case Rep Otolaryngol*; **2022**: 7232588. 35607605 10.1155/2022/7232588 PMC9124138

[16] Roland LT, Wineland AM, Leonard DS. Balloon frontal sinuplasty for intracranial abscess in a pediatric acute sinusitis patient. *Int J Pediatr Otorhinolaryngol* 2015; **79**: 432–434. 25636667 10.1016/j.ijporl.2015.01.008 PMC4867545

[17] Stamm A, Nogueira JF, Lyra M. Feasibility of balloon dilatation in endoscopic sinus surgery simulator. *Otolaryngol Neck Surg* 2009; **140**: 320–323. 10.1016/j.otohns.2008.11.034 19248935

[18] Fried MP, Sadoughi B, Gibber MJ *et al.* From virtual reality to the operating room: the endoscopic sinus surgery simulator experiment. *Otolaryngol Neck Surg* 2010; **142**: 202–207. 10.1016/j.otohns.2009.11.023 20115975

[19] Adebayo O, Jabbal M. Training the COVID generation: can we get our confidence back? *Bull R Coll Surg Engl* 2022; **104**: 10–13.

[20] Clements JM, Burke JR, Hope C *et al.* The quantitative impact of COVID-19 on surgical training in the United Kingdom. *BJS Open* 2021; **5**: zrab051. 34169311 10.1093/bjsopen/zrab051 PMC8226285

